# Generation of the pitch moment during the controlled flight after takeoff of fruitflies

**DOI:** 10.1371/journal.pone.0173481

**Published:** 2017-03-15

**Authors:** Mao Wei Chen, Jiang Hao Wu, Mao Sun

**Affiliations:** 1 School of Transportation Science and Engineering, Beihang University, Beijing, China; 2 Institute of Fluid Mechanics, Beihang University, Beijing, China; Coastal Carolina University, UNITED STATES

## Abstract

In the present paper, the controlled flight of fruitflies after voluntary takeoff is studied. Wing and body kinematics of the insects after takeoff are measured using high-speed video techniques, and the aerodynamic force and moment are calculated by the computational fluid dynamics method based on the measured data. How the control moments are generated is analyzed by correlating the computed moments with the wing kinematics. A fruit-fly has a large pitch-up angular velocity owing to the takeoff jump and the fly controls its body attitude by producing pitching moments. It is found that the pitching moment is produced by changes in both the aerodynamic force and the moment arm. The change in the aerodynamic force is mainly due to the change in angle of attack. The change in the moment arm is mainly due to the change in the mean stroke angle and deviation angle, and the deviation angle plays a more important role than the mean stroke angle in changing the moment arm (note that change in deviation angle implies variation in the position of the aerodynamic stroke plane with respect to the anatomical stroke plane). This is unlike the case of fruitflies correcting pitch perturbations in steady free flight, where they produce pitching moment mainly by changes in mean stroke angle.

## Introduction

Recently, insect flight stability has been studied widely with theoretical, experimental or numerical methods [1–6]. The natural modes of longitudinal and lateral motion are analyzed and the longitudinal unstable slow oscillatory mode [2] and lateral unstable slow divergence mode [5] are found in free flight of insects, which indicates that the flapping flight is aerodynamically unstable. Insects need to change their wing kinematics to stabilize their body posture rapidly following perturbations. Also they change the wing kinematics to perform elaborate maneuvers.

**Fig 1:**
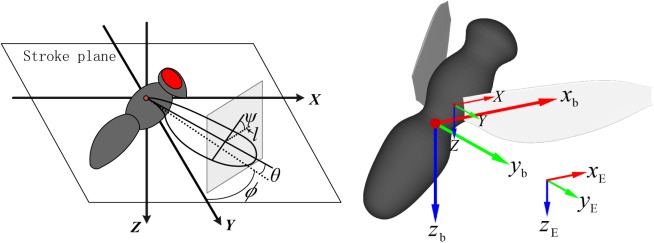
Frames to describe the wing and body kinematics: the reference frame with origin at the wing base (*X*, *Y*, *Z*). *l*, a line that is perpendicular to the wing span and parallel to the stroke plane. *ϕ*, *ψ* and *θ*: positional angle, pitch angle and deviation angle of the wing, respectively; the Earth-fixed frame (*x*_E_, *y*_E_, *z*_E_); the insect’s body-fixed frame (*x*_b_, *y*_b_, *z*_b_).

With the development of high-speed stereo videography technique, many maneuver flights and the associated wing kinematics have been studied in detail. A method given by Ellington [7] is often used to describe insect wing kinematics. In the method, the stroke plane (Fig 1) is first determined using the data of several wingbeats, and a reference frame (*X*, *Y*, *Z*) fixed with respect to the stroke plane is determined, with the origin at the wing base, the *X*-*Y* plane coinciding with the stroke plane and the *Y*-axis pointing to the side of the insect (Fig 1). Three Euler angles: positional angle (*ϕ*), stroke deviation angle (*θ*) and pitch angle (*ψ*) (see Fig 1) are used to describe the wing kinematics with respect to the stroke plane. The line connecting the wing base and wing tip is projected onto the stroke plane. The angle between the projection and the *Y*-axis is the positional angle (*ϕ*). The angle between the line connecting the wing base and tip and its projection is called the stroke deviation angle (*θ*). The line *l* in Fig 1 is perpendicular to the wing span and parallel to the stroke plane, and the pitch angle (*ψ*) is defined as the angle between the local wing chord and line *l*.*ϕ* reaches the maximum and minimum values, which are denoted as *ϕ*_max_ and *ϕ*_min_, when the wing is at the extreme position behind and in front of the body respectively. Then *Φ* (= *ϕ*_max_-*ϕ*_min_) is called as the stroke amplitude and $\bar{\phi }$[= (*ϕ*_max_+*ϕ*_min_)/2] the mean stroke angle. The angle of attack of the wing (*α*) can be given as follows: in the downstroke, *α* = *ψ*; in the upstroke, *α* = 180°- *ψ*. Here, the angle of attack is defined geometrically, but not with respect to the flow direction surrounding the wing as in most of the aeronautics literature, because it’s more convenient to describe the variation of wing kinematics during maneuver flight.

Several experimental methods to introduce aerodynamic perturbations were used to study maneuver flight of insects, such as using wind tunnel to generate unsteady turbulence [8–10] and applying impulsive torque to initiate yaw or pitch maneuvers [11],[12]. The control strategies to complete the maneuver or overcome these perturbations were studied. Ellington [7] filmed many flight sequences of different insects species in 1984. It was found that insects generated pitching moment by changing their mean stroke angle for the torque balance, and made brisk aerobatics by changing the angle of attack on either downstroke or upstroke. Cheng et al. [13] investigated the pitch rotation maneuver produced by hawkmoths, and they showed that the hawkmoths produced active pitch torque *via* changes in the angle of attack and shifted the total force vector in the opposite direction of pitching. Recently, Ristroph et al. [12] applied impulsive nose-up and nose-down torques as perturbations to fruit-flies while capturing high-speed video of their flight. Their analysis indicated that the flies generated nose-up pitch torque to overcome the perturbations by increasing the forward sweep of the wings (i.e. increasing the stroke amplitude and changing the mean stroke angle), resulting in the forward shift of the aerodynamic center. Similar result was given by Whitehead et al. [14] For lateral motion, the stroke amplitude also played an important role in controlling roll perturbations [15]. Furthermore, Ristroph and others indicated that the wing pitch angle modulations contribute to roll and yaw corrective torque in fruit flies [11],[16],[17]. The saccade maneuvers of insects have been studied widely. Fry et al. [18] recorded brisk right-angle turns of fruit-flies and their aerodynamic analysis revealed that two specific change in wing motion correlated most strongly with measured yaw torque: a backward tilt of the stroke plane and an increase in stroke amplitude; while Muijres et al. [19] found that flies lowered the angle of attack during one half-stroke while raised it on the other, thus producing an upstroke-to-downstroke imbalance in drag and thus net yaw torque. Zhang and Sun [20] studied the saccade flight of droneflies and showed that the yaw moment was mainly produced by changes in wing angles of attack (e.g. let the right wing to have a rather large angle of attack in the downstroke and a small one in the upstroke to get a right yaw torque). Greeter and Hedrick [21] studied the sideslip maneuver of moths and they found that moths used asymmetric wing angle of attack and stroke amplitude to initiate a roll, which redirected their net force vector and thus initiated lateral maneuvers. Vance et al. [22] perturbed freely-flying honey bees and stalk-eye flies with low-pressure bursts of compressed air to simulate a wind gust. Bees quickly responded to body rotations caused by gusts through bilateral asymmetry in stroke amplitude, whereas stalk-eye flies used a combination of asymmetric stroke amplitude and wing pitch angle.

We thus see that control variables used by insects in the above studies are: change in angle of attack on downstroke; change in angle of attack on upstroke; change in mean stroke angle; and change in stroke amplitude. Are there any other control variables? Is deviation angle, or flapping frequency, a control variable for some insects?

Recently, Card and others [23],[24] captured wing and body motions in detail of fruitflies performing the two types of take-off, the voluntary and the escape take-off. They got wing and body kinematic data but didn’t do dynamic analysis. We studied how the fruitflies launch themselves to the air and take off voluntarily in a recent work [25], and observed that the flies had an initial pitch rotational velocity from the takeoff jump, which can be considered as a large perturbation. Similar take-off jump was recorded by Ribak et al. in whiteflies and the pitch rotation was stopped by a damping moment produced by resting the wings backwards alongside the body to increase the aerodynamic force at the posterior tip of the body [26]. A self-righting pitch maneuver must be performed by the fruitflies after the takeoff, which has not been studied in detail. It is of great interest to find out how the maneuver is carried out and whether new control variables are used in this process.

In the present study, we film the pitch controlling flight after takeoff in fruitfiles with 3D high-speed video. Then both the time course of wing and body kinematics and the morphological data are measured. The aerodynamic forces and moments of the wings are calculated by employing the method of computational fluid dynamics (CFD). Analyzing the pitch moment and the wings motion can provide insights into how the controlling moment is generated in this flight process.

## Materials and methods

### Animals

The fruitflies of the species *Drosophila virilis* were used in our experiment, which were descended from wild-caught individuals and reared in the laboratory. The flies were 3–5 day old individuals, deprived of food for several hours prior to the start of the experiment in order to motivate flight. Only the most active ones were tested. The experiment was performed at room temperature 22–25°C.

### Experimental method

**Fig 2:**
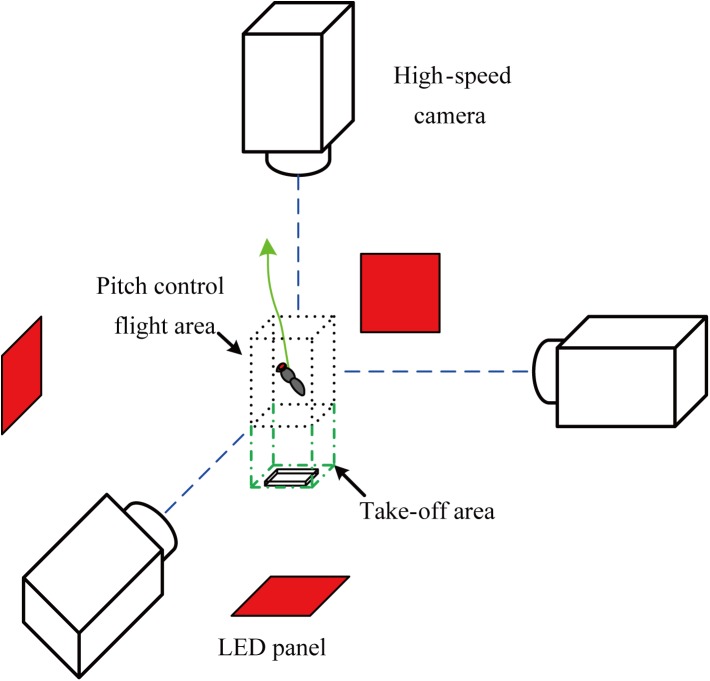
Experimental setup used to film the maneuver flights.

The high-speed filming equipment was similar with our previous work [25]. We filmed the fruitflies with three orthogonally aligned synchronized high-speed cameras (MotionXtra HG-LE, Redlake MASD, Inc., San Diego, CA, USA) in 5000 frames/s (Fig 2). In Ref. [25], we were interested in the flight from the wings starting to move until several flap cycles after the flies leaving the takeoff platform, so the cameras were focused on the area close to the top of the platform (take-off area in Fig 2). In the present study, we needed to get more pictures of the controlled flight after the takeoff. So the intersecting field of views of the three cameras was focused on the approximately 1.5×1.5×2.5cm^3^ area away from the platform (pitch control flight area in Fig 2, the height of the area is 2.5cm), and each camera view was backlit using an integrated red light emitting diode (LED). The detail setup can be found in Ref. [25].

We developed a method to extract the 3D body and wing kinematics from the filmed data and measure the morphological parameters and described it in detail in our previous work [25],[27] and others [28]-[30]. After filming, we killed the insects with ethyl acetate vapor, and cut off the wings from the body. Using a scanner (HP scanjet 4370; resolution 3600×3600 d.p.i.), we scanned the wing shape and the outline of that was used as the wing model for the following program. Also the wingless body from two perpendicular directions (the dorsoventral view and the lateral view) was scanned, and the body was replaced with the line connecting the head and end of abdomen and the line connecting the two wing hinges in the program (picture and model figure of the wing and body can be seen in Ref. [25]). A Matlab program was developed to track the 3D body and wing positions from the frames recorded by the three cameras. For each moment, the positions and orientations of the body and wings models were manually adjusted until the model image matched well with the displayed frame in three views, and taken as that of the real insect body and wings. Analysis by Mou et al. [29] showed that with this method, errors in orientation angle of the wing were less than 4°.

The wing kinematics is described using the above method and the body kinematics is determined following the convention from dynamics of flight described by Etikin and Ried [31]. Two frames of reference are used to describe the body kinematics (Fig 1): the earth-fixed frame (*x*_E_, *y*_E_, *z*_E_) and the insect’s body-fixed frame (*x*_b_, *y*_b_, *z*_b_). The *x*_E_ and *y*_E_ axes are horizontal and the *z*_E_ axis are vertical, pointing downward. For the body-fixed frame, the origin is at the center of mass (COM) of the insect’s body, the *x*_b_-*y*_b_ plane is parallel to the stroke plane. The *x*_b_ axis points forward, and the *y*_b_ axis points to the right side of the body. The displacement in *x*_E_, *y*_E_ and *z*_E_ directions of the COM of the insect’s body give the body position, denoted as Δ*x*_E_, Δ*y*_E_ and Δ*z*_E_, respectively. The three Euler angles of the insect’s body (see Ref. [31]) give the body orientation, denoted as *ψ*_b_, *θ*_b_, *ϕ*_b_,which are referred to as heading, pitch and roll angles of the body, respectively.

For flight dynamic researches, it’s significant to track the mean body motion in the time scale of several wingbeat cycles, while the sub-wingbeat wobbling motion in high frequency was often neglected. Thus, the measured body kinematic data were forward-backwards filtered using a third-order low-pass Butterworth filter, and the cut-off frequency is 90 Hz, which is much lower than the wingbeat frequency. Taking the first derivatives of the smoothed position and pitch angle data gave the velocity of the COM and body pitch angular velocity, respectively. Moreover taking the second derivatives of those smoothed data gave the acceleration of the COM and pitch angular acceleration of the body, respectively.

The total mass of the fly (*m*) was measured to an accuracy ±0.01mg after it was killed. Using the scanned picture, we can get the wing length *R* (the distance between the wing base and the wing tip) and local wing chord length directly with the accuracy better than ±2.0%. Other parameters such as wing area (*S*), mean chord length (*c*) and radius of second moment of wing area (*r*_2_), etc., were computed using the measured wing shape. We can measure the body length (*l*_b_) and distance between the wing roots (*l*_r_) from the dorsal view and the distance between the wing-base axis and the center of mass (*l*_1_) and distance between the wing-base axis and the long axis of the body (*h*_1_) from the lateral view. Following the method introduced by Ellington [32], the body was divided into hundreds of strips perpendicular to the long axis from the head to the abdomen end. The cross section of the body was taken as an ellipse and uniform density was assumed for the body. The COM and pitch moment of inertia of the body (*I*_yb_) was then estimated from those of the strips. According to Ref. [5], the influence of the wing to the pitch moment of inertia of whole insect is negligible, so *I*_yb_ is taken as the pitch moment of inertia of the whole insect.

### Calculation of the forces and moments acting on the insect

The inertia force and pitch moment about the COM acting on the insect could be calculated based on the measured data of body position and orientation, mass and pitch moment of inertia. The inertia force was given by multiplying the COM acceleration by the mass and the pitch moment was given by multiplying the angular acceleration by the pitch moment of inertia.

The aerodynamic force acting on the insect was computed using the CFD method. In order to calculate the flow around both left and right wings, the Navier-Stokes equations were solved over moving overset grids. Because the aerodynamic interaction between the body and each wing was negligibly small in low-speed flight [33]-[35], the body was neglected in the present CFD model. The numerical method was the same as that used by Sun and Yu [36]. The algorithm was based on the method of artificial compressibility developed by Rogers et al. [37], [38], which had the advantage of solving the incompressible fluid flows using the well developed methods for compressible fluid flows. With overset grids, for each wing there was an O-H type body-fitted curvilinear grid, which was generated using a Poisson solver which was based on the work of Hilgenstock [39]. In addition, there was a background Cartesian grid, which was generated algebraically, extending to the far-field boundary of the domain. Flow field data were interpolated from one grid to another at the inter-grid boundary points using tri-linear interpolation. The inflow or outflow boundary conditions were applied for the far-field boundary and impermeable wall and no-slip boundary conditions on the wing surfaces. Details could be found in Refs. [37], [38].

We used similar grid variables with our previous work [25] as follows: dimensions 75×44×60 around the wing, in the normal direction and in the spanwise direction, respectively, and 107×107×107 for the background grid; first layer grid thickness 0.0015*c* and distance from wing surface to outer boundary of wing grid 2.0*c*, where *c* was the mean chord length of wing; the background grid outer boundary from the wings 20*c*. The non-dimensional time step was 0.02. The computational code was validated in many of our previous studies (e.g. Refs. [25], [27], [34]-[36]); also a detail test of the numerical variables such as grid size, domain size, time step, was conducted in Ref. [25]. It was shown that the above values for the numerical variables were appropriate for the calculations.

## Results

Six flight sequences after voluntary takeoff performed by four fruitflies, including both male and female individuals, were filmed. The four fruitflies were denoted as FF1, FF2, FF3 and FF4, respectively. FF1 and FF2 each performed one sequence, while both FF3 and FF4 performed two sequences. Each flight sequence comprised 8–12 stroke cycles and there were 22–25 frames per stroke cycle. The morphological parameters of the insects were measured after filming and the results are given in Table 1. The following parameters are presented: *m*, mass of the insect; *R*, wing length; *S*, area of one wing; *l*_b_, body length; *h*_1_, distance from wing-root axis to long-axis of body; *l*_1_, distance from wing-root axis to body center of mass; *l*_2_, distance from anterior end of body to center of mass; *l*_r_, distance between two wing-roots; *r*_2_, radius of second moment of wing area; *I*_y,b_, pitching moment of inertia of the body.

**Table 1:** Morphological parameters of the fruitflies.

Individual	m	R	S	*l*_b_	*h*_1_/*l*_b_	*l*_1_/*l*_b_	*l*_2_/*l*_b_	*l*_r_/*l*_b_	*r*_2_/*R*	*I*_y_._b_
	(mg)	(mm)	(mm^2^)	(mm)						(mg mm^2^)
FF1 (female)	1.61	3.06	2.75	3.32	0.089	0.164	0.493	0.293	0.580	1.613
FF2 (female)	1.42	2.85	2.27	3.25	0.097	0.150	0.483	0.243	0.593	1.212
FF3 (male)	1.08	2.84	2.36	2.96	0.091	0.151	0.478	0.229	0.586	0.708
FF4 (male)	1.28	2.67	2.22	3.00	0.084	0.145	0.477	0.267	0.584	0.665

### Kinematical results

Following the method described above, the wing and body kinematics of these flight sequences were determined. For the present flight, the wingbeat frequency is approximately constant, and at the last 3–4 strokes, the flapping angles become approximately periodic in time. We determined the stroke plane using the data from the last 3–4 wingbeats, and the angle between the stroke plane and the long axis of the insect body was a constant during the flight. Then the aerodynamic force and moment were calculated with CFD method using these measured data.

Taking the flight of FF1 as an example, the original flight video sequences could be found in S1 Movie. We got the kinematical and mechanical results as follows.

**Fig 3:**
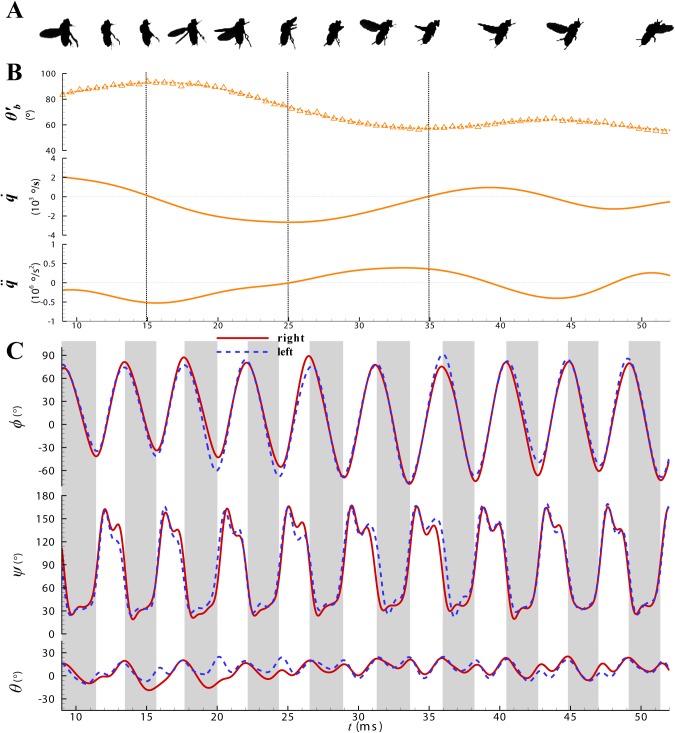
(A) Video sequences of the flight after take-off for fruitfly FF1. (B) Time histories of body pitch motion (pitch angle *θ*’_b_ = *θ*_b_+*β*_0_, angular velocity *q*_b_ and angular acceleration ${\dot{q}}_{b}$ respectively). (C) Time histories of wing flapping motion. *ϕ*, positional angle; *θ*, stroke deviation angle; *ψ*, pitch angle (*ψ* is related to the angle of attack of the wing, *α*, as: *α* = *ψ* in the downstroke and *α* = 180°-*ψ* in the upstroke). Grey bars represent the upstroke.

According to Ref. [25] and the present results, the fly typically performs two motions after takeoff, a vertical climb and a pitch rotation. The horizontal translation and the yaw angle are approximately zero. The climb velocity is approximately constant. The roll angle is within ±20°, and the average value is 0°. The roll moment is relatively small with repect to the pitch moment, because the moment of inertia about the roll axis is much smaller than that about the pitch axis. So only the pitch moment is studied in detail here. The lateral roll and yaw motions and the vertical climb have little effect on the pitch moments, and are ignored in this paper. For the wing kinematics, there are differences between the right and left wings, but these differences only effect the lateral aerodynamic moment. The pitch moment is determined by the average wing kinematics of the right and left wings, which is considered in the following content. Fig 3 shows the time histories of the wing kinematics and body pitch motion of FF1. The datasets used to produce this figure are presented in supplemental material S1 and S2 Datasets.

The fruitfly gets an initial vertical velocity and pitch-up rotational velocity when it takes off from the platform, resulting from the jump [25], and the body axis pitches up to approximately vertical after 2–3 strokes (Fig 3A, *t* = 9ms, the beginning of the first downstroke after the insect leaves the platform is denoted as 0ms). Then the pitch up angular velocity decreases and the pitch angle continues increasing to the maximal value (*θ*’_b_ = 95°, where *θ*’_b_ = *θ*_b_+*β*_0_, and *β*_0_ is the angle between the stroke plane and body axis, which is constant for each flight and 55.6° for FF1). In this time, the fly stops the pitch up rotation and the angular velocity becomes 0, corresponding to a negative nose-down pitch acceleration (*t* = 9ms-15ms, Fig 3B). The stroke amplitude is relatively small. The deviation angle *θ* is small at the end of the downstroke and the wing tip is under the stroke plane (Fig 3C). This change of deviation angle results in the tilt of the aerodynamic stroke plane. Furthermore, the wing flapping velocity and trajectory are also different.

From *t*≈15ms to *t*≈25ms, the fly starts to pitch down, and *θ*’_b_ decreases gradually. The pitch down velocity increases from 0 to a maximal value of 2500°/s, meanwhile, the nose-down angular acceleration decreases to 0 (Fig 3B). The stroke amplitude increases gradually during this period (Fig 3C). Specially, *ϕ*_max_ is approximately constant and the variety of *Φ* is as a result of variety of *ϕ*_min_ (the difference is about 30°).

After the pitch down velocity reaching its maximal value, from *t*≈25ms to *t*≈35ms, the fly continues pitching down and goes into a normal hovering orientation. The stroke plane is approximately horizontal, and *θ*’_b_ reaches approximately 60°. The angular velocity decreases to zero, and there is positive nose-up pitch acceleration during this period (Fig 3B). The stroke amplitude gets larger and has the maximal value of about 156°. The deviation angle *θ* is relatively large at the end of the downstroke comparing with the beginning of the flight and the wing tip is above the stroke plane (Fig 3C).

At the last 3–4 strokes, the fly performs a small speed climb flight, with a constant orientational angle, keeping the stroke plane horizontal. The angular velocity is approximately zero, with little oscillatory changes (Fig 3B). The stroke amplitude stays approximately constant.

Researches on the dipteran flight muscle function [40–42] indicated that the stroke angle and deviation angle are controlled by both basalar muscles, b1 and b2. The b1 is strongly correlated with the change of stroke deviation, and typically fires during the upstroke to downstroke transition, resulting in the change of deviation angle in the downstroke. Phase advance of the bl and activation of the b2 are both correlated with increased stroke amplitude, and nearly all of the modulation in stroke amplitude occurres at the ventral stroke reversal. It’s coincident with the variation of the stroke angle and deviation angle above.

In summary, the fly mainly performs a vertical climb and pitch rotation. The initial pitch up rotation velocity decreases at first and becomes negative, then decreases oscillatorily to about zero. The body pitch angle goes to about 95°, and then pitch down back to a certain angle, fitting for a steady climb flight. The stroke amplitude is small at first and increases to a certain value, while the deviation angle at the end of the downstroke at the beginning of the flight is different from that at the end. The wingbeat frequency changes little in the whole process and is about 222Hz. The body kinematics for other five flight sequences is similar (S1 Fig). The body pitch angle increases to a maximum value (varying from 60° to 100°) and then decreases to about 40°~70°.

### Results of the moment acting on the insects

**Fig 4:**
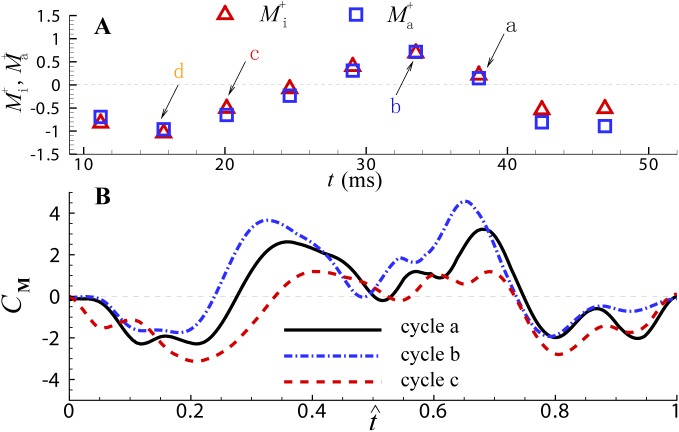
(A) The inertia pitch moment (*M*_i_) and aerodynamic pitch moment (*M*_a_) of FF1. The moments are non-dimensionalized by the *mgc*, where *c* is the mean chord length of wing: *M*_i_^+^ = *M*_i_/*mgc* and *M*_a_^+^ = *M*_a_/*mgc*. The stroke average moment of cycle a is approximately zero, and positive in cycle b, negative in cycle c and d. (B) Aerodynamic moment coefficient *C*_M_ of cycle a, b and c, and the horizontal axis normalized by the cycle period.

The time histories of inertial and aerodynamic pitch moment acting on the fly can be obtained by the method described above. Since it was the wingbeat-cycle means of the moments that affected the gross motion of the insect, we averaged the inertial and aerodynamic moments over equally sampled time points in each wingbeat cycle. The wingbeat-cycle-averaged inertial and aerodynamic pitch-moments are denoted by *M*_i_ and *M*_a_, respectively, and these moments of FF1 are shown in Fig 4A. The wingbeat cycle with almost zero pitch moment is denoted as cycle a, the one with positive pitch moment is denoted as cycle b, and two cycles with negative pitch moment are denoted as cycle c and d respectively.

After the flies taking off, there is no other force acting on the insect except for aerodynamic force and weight, so the inertial pitch moment should be equal to the aerodynamic moment (the weight does not produce moment about the COM) as shown in Fig 4A. These results also indicate that the method used to calculate the inertial and aerodynamic moment is reliable. The difference between the two moments in Fig 4A and S2 Fig is due to the measured error of the wing and body kinematics and moment of inertia, and it’s less than 0.15*mgc*.

There are nose-down moments at the beginning to stop the initial pitch up motion and the maximum moment appears at the 2nd stroke. Then the nose-down moment decreases to almost 0. Because the initial pitch up angular velocity is very large and the body long axis becomes almost vertical, a nose-down angular velocity is necessary, and a nose-up moment is generated in order to decreasing this angular velocity from *t*≈25ms to *t*≈39ms (Fig 4A). However for some other flies, when the pitch angle decreases from its maximum value, the pitch down angular velocity is not very large and also decreases smoothly. So there are no significant nose-up moments generated in these flights (S2C–S2E Fig).

## Discussion

### Generation of the pitch moment

The generation of the pitch moment of FF1 correlated with the wing kinematics is discussed as an example. Only the pitch moment and the average kinematics of the right and left wings are discussed here as mentioned above. The pitch moment is effected by the aerodynamic force parallel to the symmetric plane of the body (the *X*-*Z* plane of the frame (*X*, *Y*, *Z*)). The component along the *X* axis always points to the opposite direction to the wing stroke motion and it can be called as drag force, and the component along the *Z* axis points upward perpendicularly to the stroke plane and it is called as lift force. Changes in either the aerodynamic force or the moment arm to the COM will affect the pitch moment.

As shown in Fig 4A, the moment is almost 0 in cycle a, so this cycle can be considered the same as a flap cycle when the fly is hovering. There is a positive nose-up moment in cycle b, a negative nose-down moment in cycle c and a larger negative nose-down moment in cycle d. The aerodynamic moment coefficient *C*_M_ is shown in Fig 4B (time histories of the aerodynamic moment coefficient is given in S3 Dataset), which is defined as *C*_M_ = *M*_a_/(0.5*ρU*^2^*Sc*) (in which *M*_a_ is the aerodynamic pitch moment, *ρ* is the air density, *S* is the wing area, *c* is the mean wing chord, *U* is the reference velocity, *U* = 2*Φr*_2_*n*). The horizontal axis is normalized by the cycle period, and it’s the beginning of the downstroke at $\hat{t}$ = 0 and the end of the upstroke at $\hat{t}$ = 1. It can be seen that for cycle a, in the downstroke it’s nose-down moment at the first half and nose-up moment at the second half, and the average value is approximate 0 in the whole downstroke; it’s also the same for the upstroke, and the difference is that it’s nose-up moment at the first half and nose-down moment at the second half. Compared with cycle a, for cycle b, the moment is larger in the whole downstroke and the beginning of the upstroke (0.5<$\hat{t}$<0.7). It’s almost the same at 0.7<$\hat{t}$<0.9 and a little larger at the end of upstroke (0.9<$\hat{t}$<1). For cycle c, the moment is smaller in the whole period (Fig 4B).

**Fig 5:**
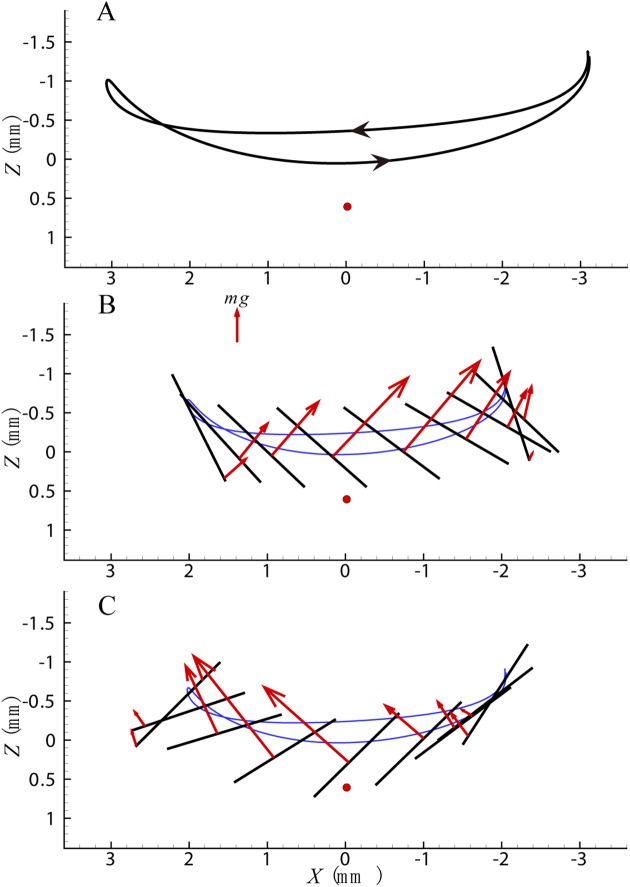
(A) Wing tip trajectory of cycle a. The red solid circle denotes the body mass center. (B) Aerodynamic force in the downstroke of cycle a. The reference vector denotes the magnitude of the insect weight. (C) Aerodynamic force in the upstroke of cycle a.

The wing tip trajectory and aerodynamic force for cycle a are shown in Fig 5, and it’s the *X*-*Z* plane of the frame (*X*, *Y*, *Z*) shown in the figure, the red dot is denoted as the COM of the body. The wing tip trajectory is shown in Fig 5A and the arrow stands for the move direction of the wing (from right to left it denotes downstroke, and from left to right it is upstroke). The aerodynamic force in downstroke and upstroke is shown in Fig 5B and 5C respectively. For simplicity, it is assumed that the force acts on the wing surface at 66% of the wing length from the root [43]. The cross section of the wing at this position at 9 temporally equidistant points within the down or up stroke (the tilted angle stands for the attack angle of the wing) is shown in the figure. The force is projected to the plane and the acting point can be determined according to the calculated aerodynamic force and moment (the arrow indicates the force direction and the length of the line indicates the magnitude of the force). Then it can be seen from the figure directly how the aerodynamic moment changes with the aerodynamic force.

It can be seen that the wing tip trajectory is shallow U-shaped curve in the downstroke and deep U-shaped one in the upstroke (Fig 5A), which is similar to that of a hovering fruitfly [30], [44]. This is another evidence that supports the assumption that the kinematic and aerodynamic force of ‘cycle a’ is the same as that in hovering flight.

The aerodynamic force is approximately perpendicular to the wing plane (Fig 5B and 5C). It has maximum value in the middle of downstroke (Fig 5B), when the moment arm is small, resulting in a small moment. A nose-down moment in the first half and nose-up moment in the second half of downstroke are generated (Fig 5B, 0<$\hat{t}$<0.5), and a large aerodynamic force and nose-up moment are generated by a fast downward motion of the wing with large attack angle (Figs 4B and 5C, 0.5<$\hat{t}$<0.71). However, the moment arm is small when the force reaches its maximum value, and the moment is also very small (Figs 4B and 5C, 0.71<$\hat{t}$<0.75). At the end of upstroke the wing moves upward resulting in a relatively small force, and a nose-down moment is generated (Figs 4B and 5C, 0.75<$\hat{t}$<1). It should be point out that the drag force always generates nose-up moment during downstroke and nose-down moment during upstroke and the deviation angle can affect the arm of the drag force by raising or lowering the wing. While the lift force generates nose-up moment in front of the COM and nose-down moment at the back of the COM and the stroke angle can affect the arm of the lift force.

**Fig 6:**
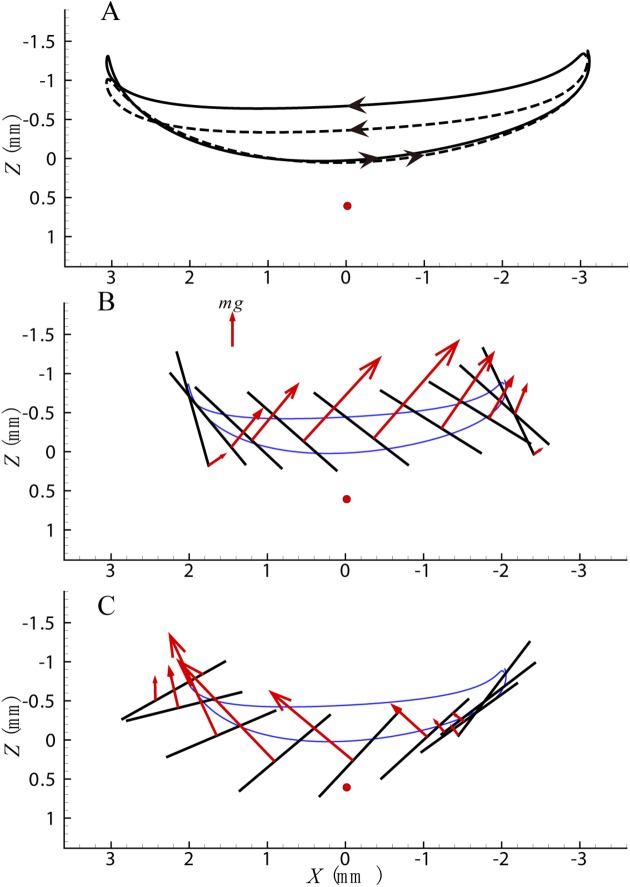
(A) Wing tip trajectory of cycle b. The dash line indicates the wing tip trajectory of cycle a. (B) Aerodynamic force in the downstroke of cycle b. (C) Aerodynamic force in the upstroke of cycle b.

For cycle b, when a nose-up moment is generated, the wing tip trajectory and aerodynamic force are shown in Fig 6 (the dash line in Fig 6A indicates the wing tip trajectory of cycle a). Compared with cycle a, in the downstroke of cycle b, the deviation angle is larger, and the wing tip trajectory is above that of cycle a. Hence the arm of the drag force is larger resulting in a larger nose-up moment (Fig 4B, 0<$\hat{t}$<0.5). At the beginning of the upstroke the wing position is higher and the magnitude of the downward motion is larger, resulting in a larger force and nose-up moment (Fig 4B, 0.5<$\hat{t}$<0.71). The wing trajectory is almost the same at the rest of the upstroke and the difference of the aerodynamic force is small (Fig 6A). Meanwhile the stroke angle changes a little and the acting position of the lift force moves forward slightly.

**Fig 7:**
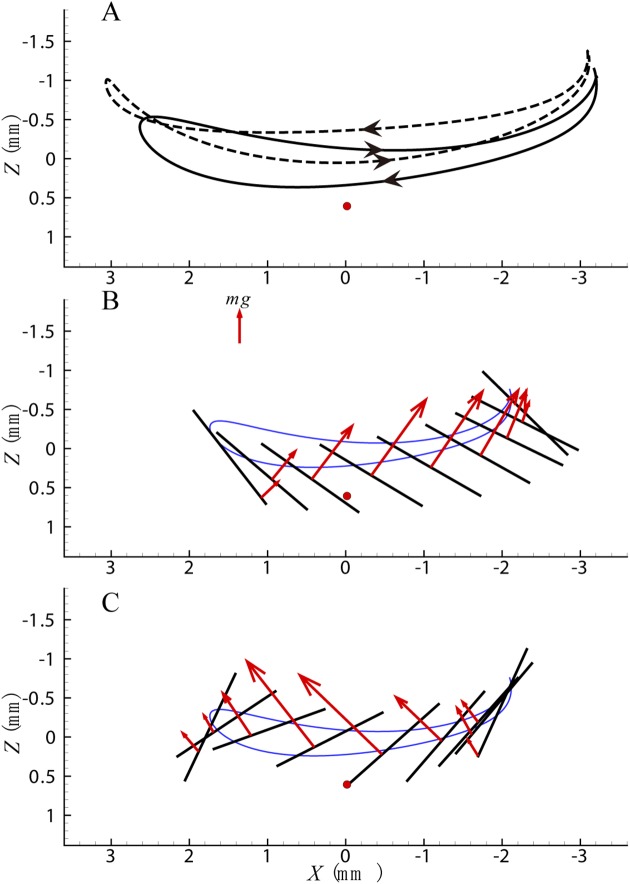
(A) Wing tip trajectory of cycle c. The dash line indicates the wing tip trajectory of cycle a. (B) Aerodynamic force in the downstroke of cycle c. (C) Aerodynamic force in the upstroke of cycle c.

Oppositely, for cycle c when a nose-down moment is generated, the wing tip trajectory and aerodynamic force are shown in Fig 7. Compared with cycle a, in the downstroke of cycle c, the deviation angle is smaller, and the wing tip trajectory is under that of cycle a. Hence the arm of the drag force is smaller (Fig 7A and 7B). At the beginning of the upstroke the wing position is lower followed by a small magnitude of downward motion, which decreases the aerodynamic force (Fig 7A and 7C). All of these result in a larger nose-down moment. Also the deviation angle in the upstroke is a little larger, causing larger moment arm of the drag and larger nose-down moment, but this change is much less significant than that in the downstroke. Moreover, the stroke amplitude decreases a little because of the change of position angle at the end of the downstroke, and the acting position of the lift force in the whole wingbeat moves backwards resulting in a larger nose-down moment.

**Fig 8:**
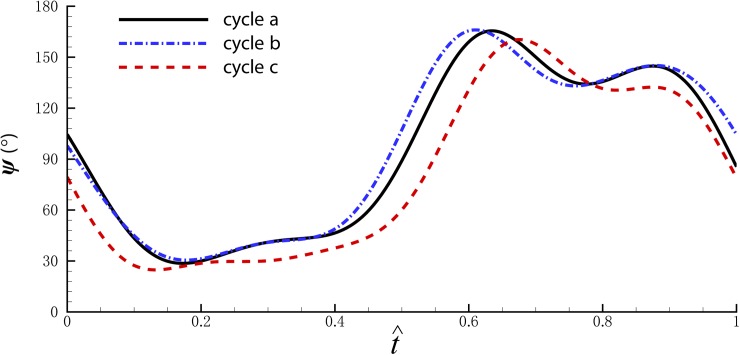
The pitch angle of cycle a, b and c. The horizontal axis is normalized with wingbeat cycle.

Although the aerodynamic force can be affected by the stroke and deviation angles, it is more sensitively correlative to the angle of attack. The pitch angles *ψ* of cycle a, b and c are shown in Fig 8. The angle of attack of the wing (*α*) can be given as follows: in the downstroke, *α* = *ψ*; in the upstroke, *α* = 180°- *ψ*. Generally, for cycle b, the angle of attack is a little larger in the downstroke, causing a larger drag force and increasing the nose-up moment. At the beginning of the upstroke, the wing starts to flap backward earlier than cycle a, and the angle of attack reverses synchronously, leading to the increase of the lift peak resulting from the acceleration of the stroke and deviation angle, which also increases the nose-up moment. The angle of attack changes oppositely for cycle c when a nose-down moment is generated.

Specially, there is damping moment opposite from the pitching direction. Taking cycle c for example, the pitch angular velocity is about -2000°/s (Fig 3), and the nose-up damping moment is generated. We fix the body and calculate the pitch moment without any body motion, the average pitch moment of cycle a, b and c changes from 0.109, 0.708 and -0.723 to 0.124, 0.688 and -0.988 respectively. It can be seen that in cycle a and b, the pitch angular velocity is much smaller (Fig 3), thus does not have too much effect on the pitch moment.

The generation of the pitch moment in other wingbeat cycles and other flights is similar. The wing tip trajectory of a cycle generating nose-up moment for FF2 and FF3 are shown in S3 Fig respectively, also the data when nose-down moment is generated for the other five flights are shown in S4 Fig. The deviation angle decreases in downstroke in cycle b and increases in cycle c. While in the upstroke, it increases a little but not as significantly as in the downstroke, and even has little differences in some flight sequences (S4 Fig).The stroke angle varies at the end of the downstroke and changes the stroke amplitude and mean stroke angle. The wing pitch angle of all these cycles for the other flights also changes the same as FF1 described above (S5 Fig).

In summary, the pitch moment can be generated by changing either the aerodynamic force parallel to the body symmetric plane or the arm of the aerodynamic force. When a nose-up moment is generated, the angle of attack increases in the downstroke and reverses earlier at the beginning of the upstroke to change the aerodynamic force. The deviation angle increases during the downstroke, resulting in a larger arm of drag force and aerodynamic force at the beginning of upstroke. The stroke angle extends forward slightly, shifting the acting position of the lift force forward. Conversely, in the cycle generating nose-down moment, the angle of attack decreases in the downstroke and reverses later at the beginning of the upstroke. The deviation angle decreases, thus the arm of drag force and aerodynamic force at the beginning of upstroke both decrease. Meanwhile, the stroke angle changes remarkably for some cycles to shift the acting position of the lift force backward.

### Effect of wing kinematic angles on the pitch moment

Several researches on the fruitflies pitch motion showed that the generation of the pitch moment is ascribed to the shift of mean stroke angle [12],[14]. It’s clear that the shift of mean stroke angle leads to the change of moment arm of the lift to COM and varies the pitch moment. While in present study, taking FF1 for example, the deviation angle changes dramatically for cycle b and c, changing the moment arm of the drag; this might have a great influence on the generation of the pitch moment. Meanwhile, the pitch angle or the angle of attack affects the aerodynamic force sensitively; this also might affect the pitch moment.

**Fig 9:**
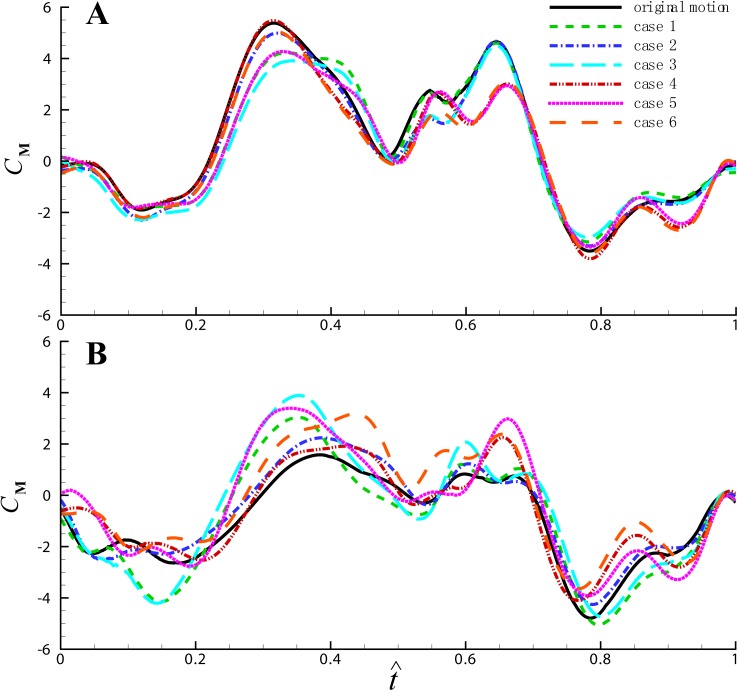
The time histories of the pitch moment coefficients in a cycle for each case. The horizontal axis is normalized with wingbeat cycle. (A) cycle b; (B) cycle c.

In order to examine the influence of three wing kinematic angles: the stroke angle, the deviation angle and the angle of attack, we replaced one or two of these three angles of cycle b and c by those of cycle a, and recalculated the aerodynamic force and moment with these virtual wing kinematics (the body motion is set to zero to get rid of the damping moment). The time histories of the pitch moment coefficients in a cycle are shown in Fig 9 and the cycle-average values are shown in Table 2. In the table, Case 1 is the case in which the stroke position angle *ϕ* of cycle b (or c) is replaced by that of cycle a; Case 2 is the case in which *θ* is replaced by that of cycle a, and so on; $\Delta {\bar{C}}_{M}$ denotes the difference in cycle-average moment coefficients between each case and cycle a. As seen in the table, with the original wing motion, $\Delta {\bar{C}}_{M}$ is positive (0.564) in cycle b and negative (-1.112) in cycle c, which means it’s nose-up and nose-down moment in cycle b and c respectively; when one or two of the wing angles are replaced, the magnitude of $\Delta {\bar{C}}_{M}$ is reduced generally.

**Table 2:** Difference in cycle-average moment coefficients compared with cycle a.

	Cycle b		Cycle c	
	$\Delta {\bar{C}}_{M}$	Percentage of change in $\Delta {\bar{C}}_{M}$	$\Delta {\bar{C}}_{M}$	Percentage of change in $\Delta {\bar{C}}_{M}$
Original wing motion	0.564		-1.112	
Case 1: *ϕ*	0.458	-18.8%	-1.113	0.1%
Case 2: *θ*	0.355	-37.1%	-0.816	-26.6%
Case 3: *ψ*	0.211	-62.6%	-0.807	-27.4%
Case 4: *ϕ&θ*	0.268	-52.5%	-0.815	-26.7%
Case 5: *ϕ&ψ*	0.177	-68.6%	-0.528	-52.5%
Case 6: *θ&ψ*	0.100	-82.3%	-0.291	-73.8%

As discussed above, the pitch moment is influenced by change in the moment arm, which is mainly due to the variations in the stroke and deviation angles. It can be seen from Table 2 that when the stroke angle is replaced, $\Delta {\bar{C}}_{M}$ decreases by 18.8% for cycle b and has almost no change for cycle c, while when the deviation angle is replaced, $\Delta {\bar{C}}_{M}$ reduces by 37.1% and 26.6% respectively. The effect of the deviation angle is more significant than the stroke angle. The influence of these two angles can be linearly superimposed (case 4 in Table 2), and this is because the stroke angle affects the moment arm of the lift and the deviation angle affects the moment arm of the drag.

The angle of attack affects the pitch moment significantly by varying the aerodynamic force. When the angle of attack is replaced, $\Delta {\bar{C}}_{M}$ decreases by 62.6% and 27.4% for cycle b and c respectively. Furthermore, the angle of attack coordinates with the stroke angle and deviation angle, and changes both the aerodynamic force and moment arm to affect the pitch moment. Taking cycle c as example, $\Delta {\bar{C}}_{M}$ decreases by 52.5% when the stroke angle and angle of attack are both replaced (case 5 in Table 2), while it has little change when only the stroke angle is replaced and reduces by 27.4% when only the angle of attack is replaced.

We also computed the angular displacement of pitch during one cycle with same initial angular velocity at the beginning using the wing kinematics of each case in Table 2 and cycle a. The difference in the angular displacement of pitch between each case and cycle a Δ*θ*_b_ is shown in Table 3. We can see that the changes in Δ*θ*_b_ of each case are similar to that of $\Delta {\bar{C}}_{M}$ in Table 2. The influence of the stroke angle on the angular displacement is less significant than that of the deviation angle and angle of attack.

**Table 3:** Difference in the angular displacement of pitch compared with cycle a.

	Cycle b		Cycle c	
	Δ*θ*_b_ (°)	Percentage of change in Δ*θ*_b_	Δ*θ*_b_ (°)	Percentage of change in Δ*θ*_b_
Original wing motion	3.6		-6.2	
Case 1: *ϕ*	2.4	-33.3%	-6.1	-1.6%
Case 2: *θ*	2.1	-41.7%	-4.8	-22.6%
Case 3: *ψ*	0.7	-80.6%	-4.6	-25.8%
Case 4: *ϕ&θ*	2.5	-30.6%	-4.6	-25.8%
Case 5: *ϕ&ψ*	1.4	-61.1%	-2.7	-56.5%
Case 6: *θ&ψ*	1.2	-66.7%	-2.5	-59.7%

It can be seen that although all the wing kinematic angles affect the pitch moment generation, the stroke angle is less important in the take-off flights. Specially, the maximum of stroke angle is almost constant (approximately 80°) and the flies vary the minimum of stroke angle to change the stroke amplitude and mean position. Because the minimum stroke angle of cycle a is particularly large (more than 70°), the mean stroke angle can’t decrease too much. Meanwhile, when the minimum increases and the mean stroke angle shifts backward (cycle c), the stroke amplitude also reduces. As a result, the lift force reduces and can’t balance the weight of the insects. So these disadvantages limit the usage of this control strategy of changing the stroke angle in takeoffs.

## Conclusion

After takeoff, a fruit-fly has a large pitch-up angular velocity owing to the takeoff jump and the fly controls its body attitude by producing a large pitching moments. It is found that the pitching moment is produced by changes in both the aerodynamic force and the moment arm. The change in the aerodynamic force is mainly due to the change in angle of attack. The change in the moment arm is mainly due to the change in the mean stroke angle and deviation angle, and the deviation angle plays a more important role than the mean stroke angle in changing the moment arm (note that change in deviation angle implies variation in the position of the aerodynamic stroke plane with respect to the anatomical stroke plane). This is unlike the case of fruitflies correcting pitch perturbations in steady free flight, where they produce pitching moment mainly by changes in mean stroke angle.

## Supporting information

S1 Movie Original video sequences of the controlled flight of FF1. (MPG)

S1 Dataset Time histories of body orientation angle, angular velocity and angular acceleration. The quantities in each column are *t* (ms), body orientation angle *ψ*_b_, *θ*_b_, *ϕ*_b_ (radian), angular velocity along body-fixed *x*_b_, *y*_b_, *z*_b_ axes (radian/s), angular acceleration along body-fixed *x*_b_, *y*_b_, *z*_b_ axes (radian/s^2^), respectively.
(DAT)

S2 Dataset Time histories of wing kinematic angles. The quantities in each column are *t* (ms), wing positional angle (*ϕ*) for right and left wings, stroke deviation angle (*θ*) for right and left wings and pitch angle (*ψ*) for right and left wings, respectively. The angles are in the unit radian.
(DAT)

S3 Dataset Time histories of the aerodynamical pitch moment coefficient. The quantities in the two columns are *t* (ms) and non-dimensional pitch moment coefficient *C*_M_, respectively.
(DAT)

S1 Fig Time histories of the body pitch angle *θ*’_b_ = *θ*_b_+*β*_0_ of the other five flights. (A) FF2. (B) FF3. (C) FF3a. (D) FF4. (E) FF4a.
(TIF)

S2 Fig Time histories of the inertia pitch moment (*M*_i_) and aerodynamic pitch moment (*M*_a_) of the other five flights. Cycle a, b and c denote the wingbeat cycle with zero moment, nose-up moment and nose-down moment respectively. (A) FF2. (B) FF3. (C) FF3a. (D) FF4. (E) FF4a.
(TIF)

S3 Fig Wing tip trajectory of cycle b when the nose-up moment is generated for other flights. The dash line corresponds to the cycle with zero pitch moment. (A) FF2. (B) FF3.
(TIF)

S4 Fig Wing tip trajectory of cycle c when the nose-down moment is generated for the other five flights. The dash line corresponds to the cycle with zero pitch moment. (A) FF2. (B) FF3. (C) FF3a. (D) FF4. (E) FF4a.
(TIF)

S5 Fig Wing pitch angle *ψ* of cycle a, b and c in the other five flights. (A) FF2. (B) FF3. (C) FF3a. (D) FF4. (E) FF4a.
(TIF)
