# Supplementary figures and images for: Generation of the pitch moment during the controlled flight after takeoff of fruitflies
Source: PLoS One. 2017 Mar 15;12(3):e0173481. doi: 10.1371/journal.pone.0173481 (PMC5351871; doi:10.1371/journal.pone.0173481)

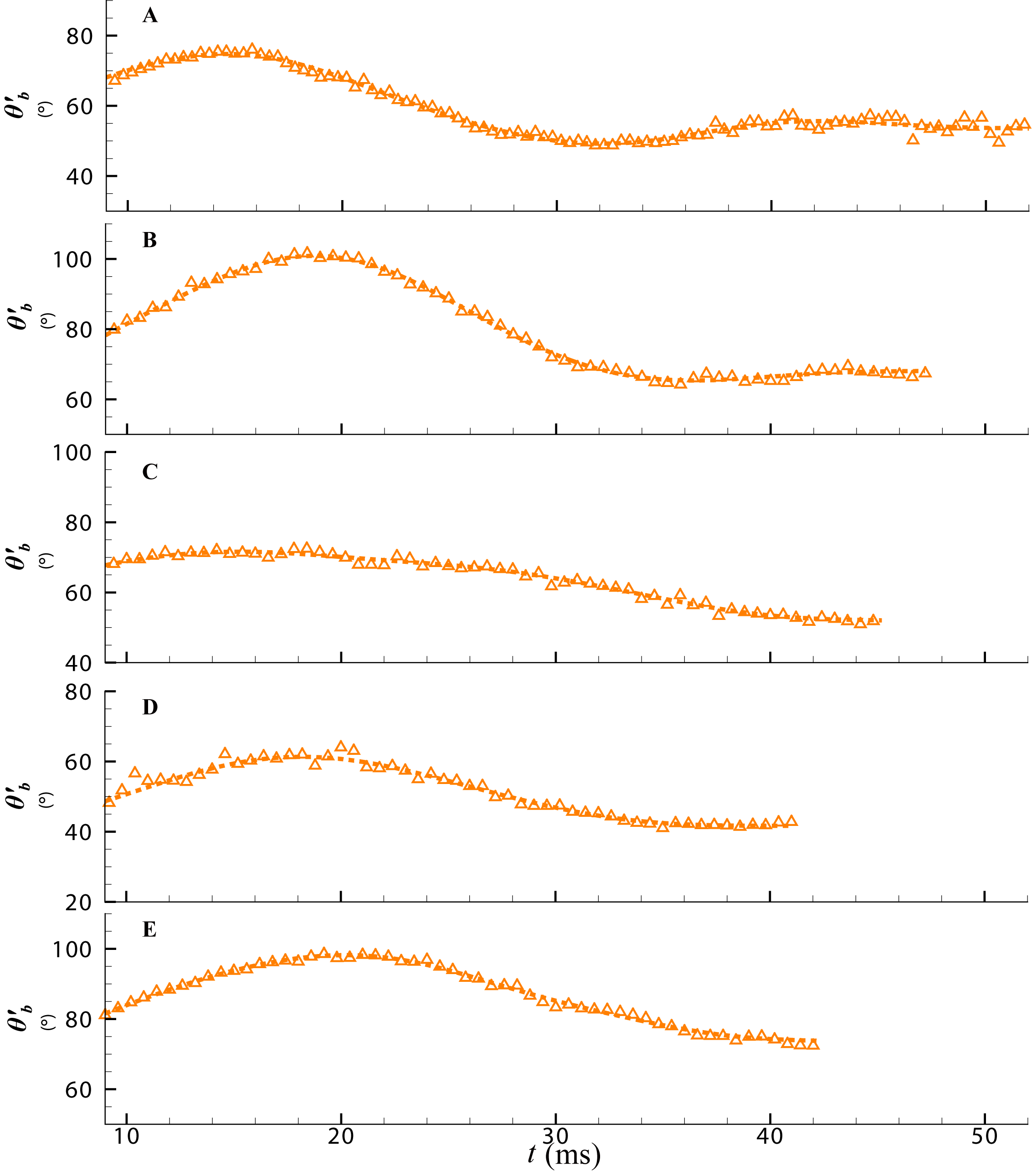

Supplement: S1 Fig — (A) FF2. (B) FF3. (C) FF3a. (D) FF4. (E) FF4a. (TIF) [file pone.0173481.s005.tif]

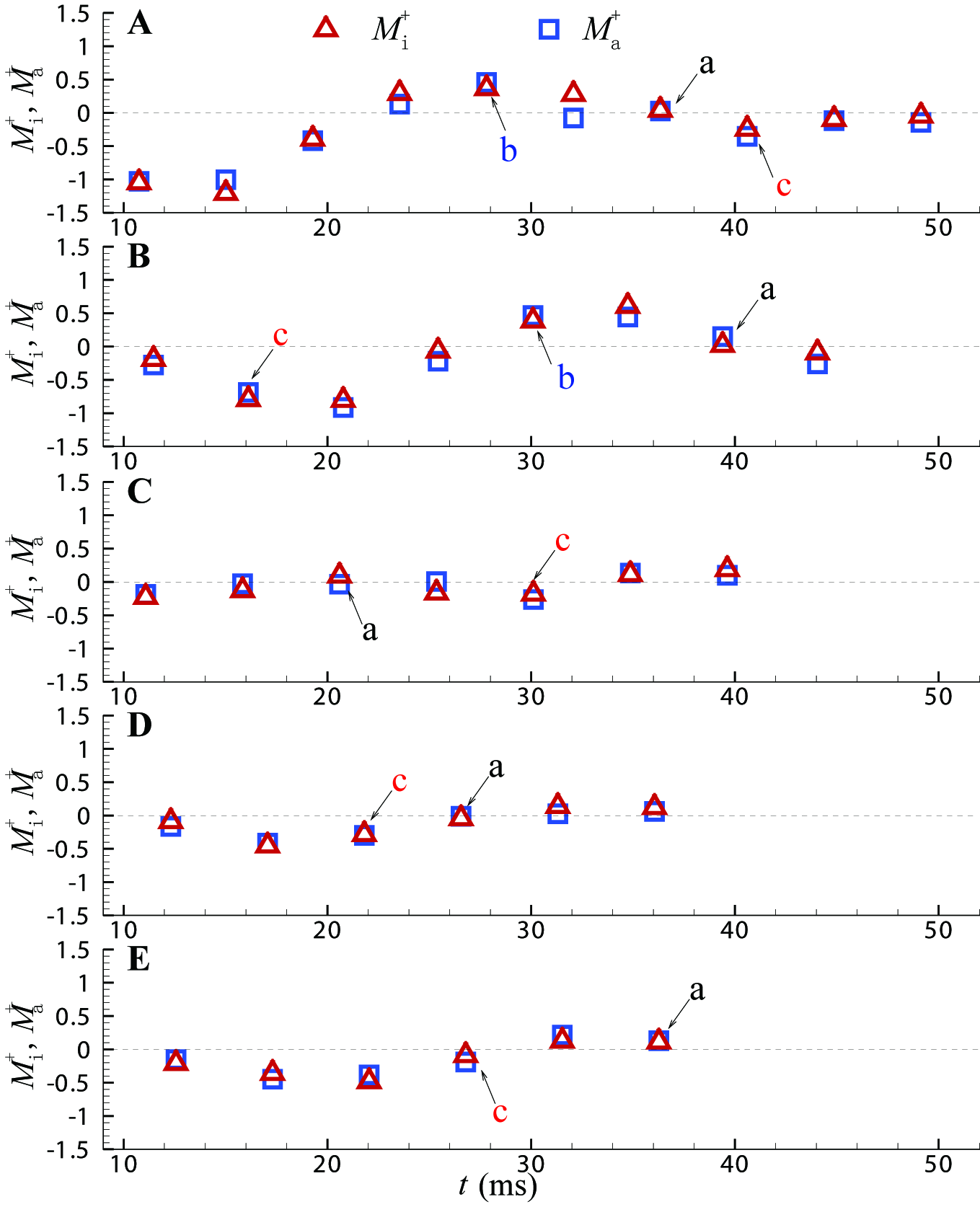

Supplement: S2 Fig — Cycle a, b and c denote the wingbeat cycle with zero moment, nose-up moment and nose-down moment respectively. (A) FF2. (B) FF3. (C) FF3a. (D) FF4. (E) FF4a. (TIF) [file pone.0173481.s006.tif]

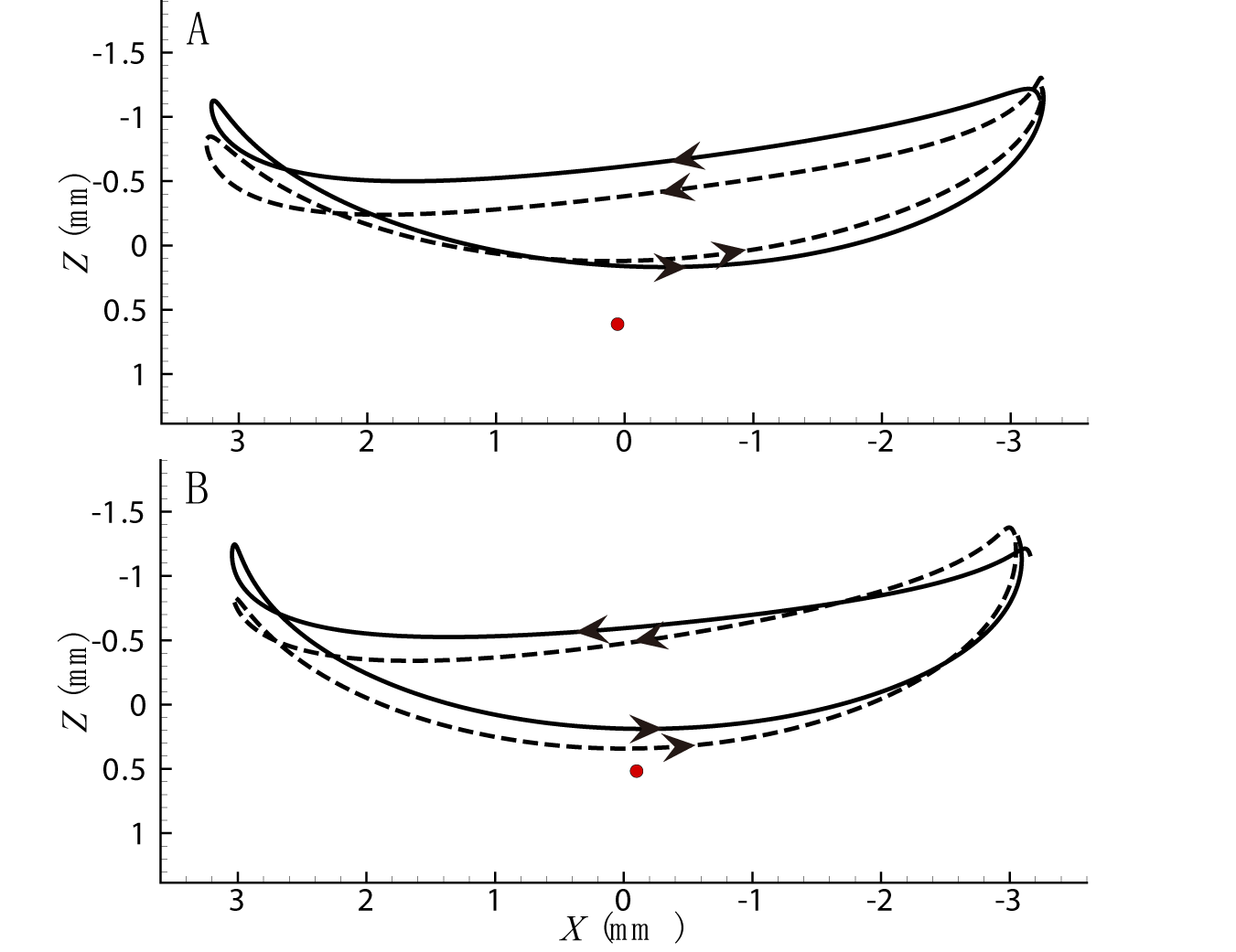

Supplement: S3 Fig — The dash line corresponds to the cycle with zero pitch moment. (A) FF2. (B) FF3. (TIF) [file pone.0173481.s007.tif]

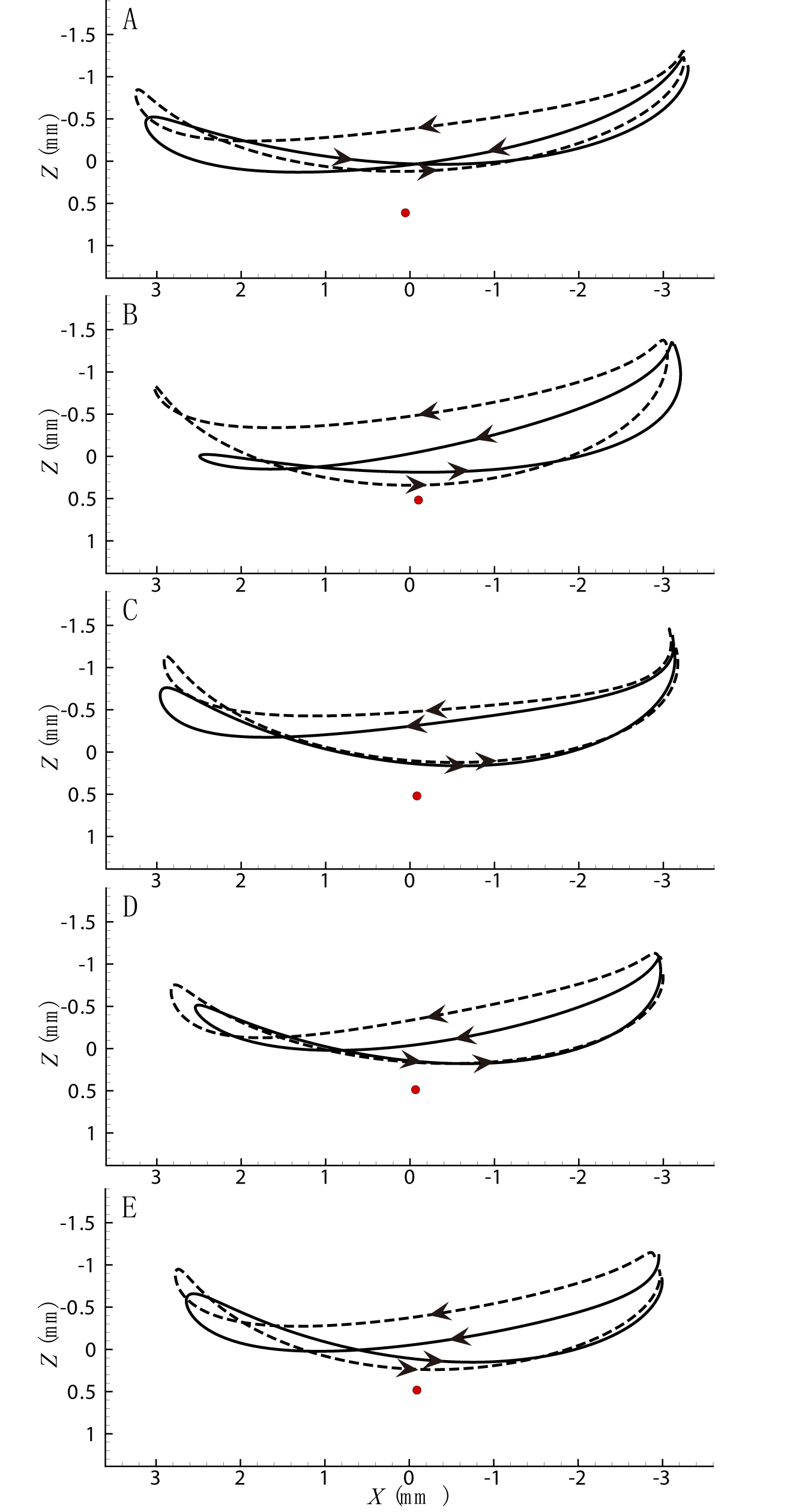

Supplement: S4 Fig — The dash line corresponds to the cycle with zero pitch moment. (A) FF2. (B) FF3. (C) FF3a. (D) FF4. (E) FF4a. (TIF) [file pone.0173481.s008.tif]

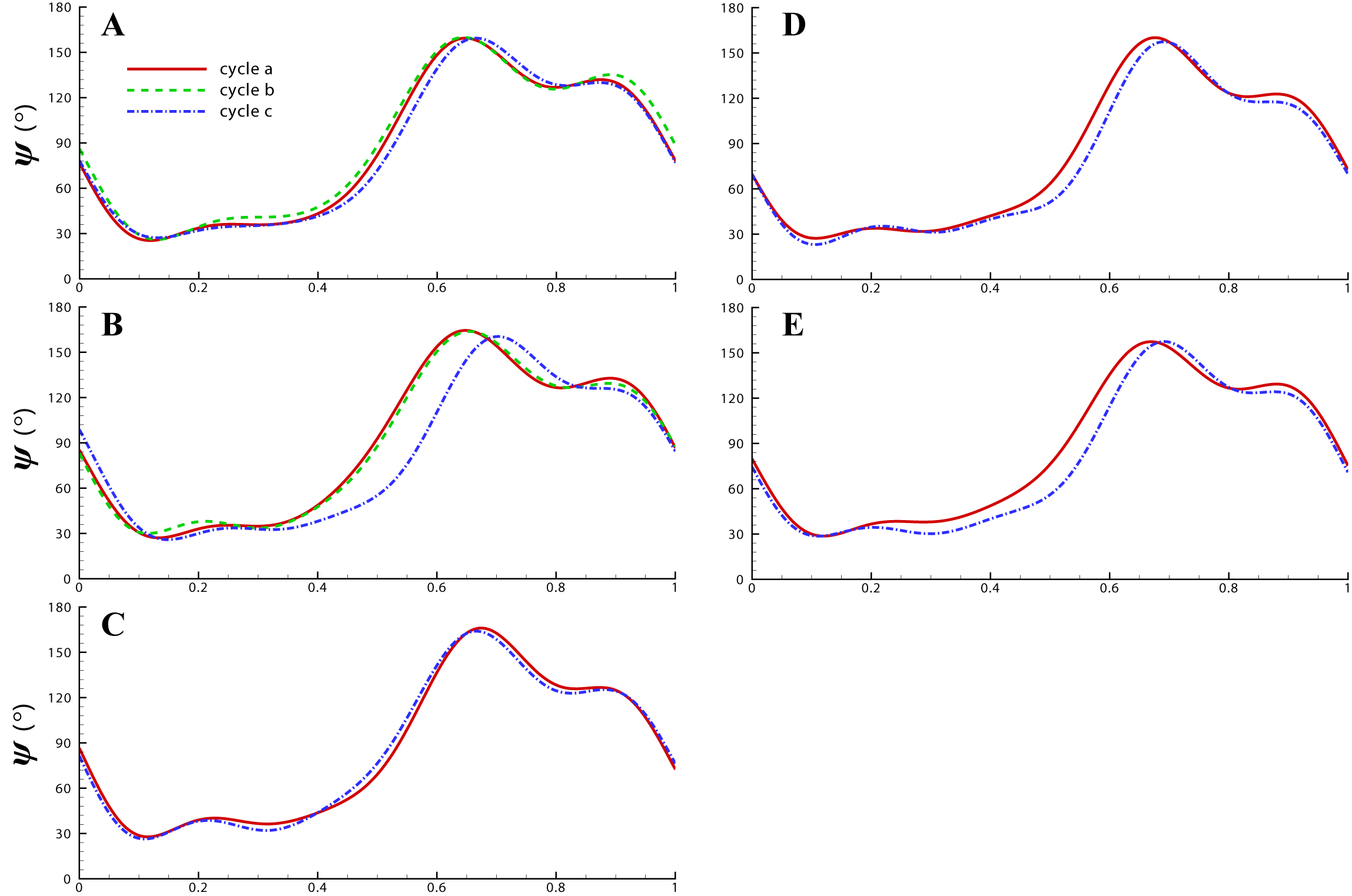

Supplement: S5 Fig — (A) FF2. (B) FF3. (C) FF3a. (D) FF4. (E) FF4a. (TIF) [file pone.0173481.s009.tif]
